# The general practitioner workforce crisis in England: a qualitative study of how appraisal and revalidation are contributing to intentions to leave practice

**DOI:** 10.1186/s12875-016-0489-9

**Published:** 2016-07-20

**Authors:** Jeremy Dale, Rachel Potter, Katherine Owen, Jonathan Leach

**Affiliations:** Primary Care, Warwick Medical School, Coventry, CV4 7AL UK; Research Fellow, Warwick Medical School, Coventry, CV4 7AL UK; Teaching Fellow, Warwick Medical School, Coventry, CV4 7AL UK; Davenal House Surgery, Bromsgrove, B61 0DD UK

**Keywords:** Family practice, General practice, Workforce, Revalidation, Appraisal, Retirement

## Abstract

**Background:**

The general practice (GP) workforce in England is in crisis, with declining morale and job satisfaction, increasing early retirement and declining interest in training to become a GP. We recently reported on factors that are influencing this, with appraisal and revalidation emerging as an unexpected finding; 28.6 % of GPs stating an intention to leave general practice within the next 5 years included this as ‘very important’ or ‘important’ to their decision. In this study we undertook a secondary analysis to identify how the experience of appraisal and revalidation might be influencing intentions to leave general practice.

**Methods:**

Qualitative analysis of free text comments made by GPs in a survey of career intentions. All comments that included mention of appraisal or revalidation were extracted. Emergent themes were identified and a coding framework devised.

**Results:**

Forty-two participants made comments that related to appraisal and revalidation. Compared to all 1192 participants who completed the main survey, they were older (76.2 % compared to 46.2 % aged 50 years and older), with more years’ general practice experience (80.0 % compared to 48.0 % with >20 years’ experience) and more likely to state an intention to retire within 5 years (72.2 % compared to 41.9 %).

Key themes were appraisal and revalidation as: a bureaucratic, inflexible exercise that added to an already pressured workload; an activity that has little educational value, relevance to professional development or quality of care; and an issue that contributes to low morale, work-related distress and intentions to leave general practice. Revalidation was depicted as a cumbersome tick-box exercise that had little to do with quality of care or protecting patients. There were no comments that countered these negative views.

**Conclusions:**

While the representativeness of these comments to the experience of GPs as a whole cannot be judged, it is likely that that they reflect the concerns of GPs whose experience of appraisal and revalidation is influencing their intention to leave general practice. Through its impact on GP morale and burnout, the current appraisal and revalidation system in England appears to be contributing to the workforce crisis. The findings indicate that the appraisal system may be in urgent need of re-design to increase its relevance to individual GPs’ experience and seniority, clinical activities being undertaken and professional development needs.

## Background

General practitioners (GPs) in England are experiencing unmanageable work-related stress, with increasing numbers considering early retirement or relocation [1]. We recently reported findings from a regional survey in which 82.0 % of responding GPs stated that they intend to leave general practice, take a career break and/or reduce clinical hours of work within the next 5 years [2]; key findings are presented in Table 1. Numerous factors were identified as contributing to workload pressure and professional burnout, including volume of workload, intensity of workload, time spent on ‘unimportant tasks’, and declining job satisfaction. One issue that emerged in free text responses was GPs’ experience of appraisal and revalidation. GPs who stated an intention to leave general practice within the next 5 years described revalidation as either a ‘very important’ (17.9 %) or ‘important’ (10.7 %) issue that was contributing to their decision.

**Table 1:** Key findings from the West Midlands GP workforce survey [2]

Of 1,192 GPs who participated, 978 (82.0 %) stated that they intend to leave general practice, take a career break and/or reduce clinical hours of work within the next 5 years. This included 488 (41.9 %) who intend to leave practice, and almost a quarter (279; 23.2 %) intending to take a career break. Only 67 (5.6 %) planned to increase their hours of clinical work. For participants planning to leave practice, the issues that most influenced intentions were volume and intensity of workload, time spent on “unimportant tasks”, introduction of 7-day working and lack of job satisfaction. Four hundred fifty-five participants provided free-text responses (39128 words in total). The main themes were the cumulative impact of work-related pressures, the changing and growing nature of the workload, and the consequent stress. Sub-themes included the impact on GP workload of the following: growth in patient expectations and demand; GP recruitment and retention difficulties; burgeoning administration and bureaucracy; growth in additional roles, responsibilities and time involved in meetings; transfer of work from secondary care; increasing complexity and chronic ill health; revalidation and regulatory assessment; and the introduction of 7-day working in general practice. Reducing workload intensity, workload volume, administrative activities, with increased time for patient care, no out-of-hour commitments, more flexible working conditions and greater clinical autonomy were identified as the most important requirements to address the workforce crisis. In addition, incentive payments, increased pay and protected time for education and training were also rated as important.	

GP appraisal became compulsory in England in 2004 following proposals made in “Supporting Doctors, Protecting Patients” [3]. It was initially introduced as a peer-led, formative and confidential process intended to enable self-reflection and professional development. Reported benefits included enhanced learning, confidence in practice and improved patient care [4–6]. However, some GPs found appraisal unhelpful, citing the time taken to complete documentation for a process which they felt was politically motivated and lacked educational benefit [5,6].

Revalidation was introduced in 2012 for doctors in the UK, with all those on the General Medical Council (GMC) register becoming required to revalidate every 5 years by demonstrating through their annual appraisals that *“… they are practising in accordance with the GMC guidance Good Medical Practice across the whole of their scope of practice* [7]. This had been preceded by two decades of debate in which the feasibility, strengths and weaknesses of different approaches to revalidation, also referred to as reaccreditation or recertification, were debated [8–10]. Numerous concerns were voiced, including that the proposed process would be time-consuming, costly and encourage ‘gaming’ and evasion in order to avoid personal weaknesses from emerging that might risk successful completion of the process [11–15].

Little research has been undertaken into the effectiveness of appraisal and revalidation on maintaining quality of care, the costs that are involved, or its impact on doctors’ job satisfaction. In this study, we undertook a secondary analysis of free text comments from the previous survey [2] in order to explore how GPs describe the experience of appraisal and revalidation, and how this may be influencing their intentions to remain active in the GP workforce. We undertook this analysis cognisant of the methodological considerations associated with use of free text comments that have been discussed elsewhere [16].

## Method

Between December 2014 and January 2015 we conducted an online survey to explore the career intentions of GPs working in the West Midlands region of England [2]. The survey covered work-related morale and job satisfaction, career intentions and, for participants who reported that they intended to leave general practice or take a career break within the next 5 years, factors that had influenced their decision or might reverse this intention (Table 1). Participants were not asked to provide personal information, such as name, address or practice, and ticked a box to confirm that they understood and gave consent for their responses to be included in research publications.

The survey included free text space associated with the following items for participants to clarify their responses and to identify any issues inadequately covered by the questionnaire: *Which factors are contributing to your decision about when to leave/retire from general practice? Please indicate the extent to which the following factors might encourage you to remain in general practice? Which, if any, of the following might encourage you to continue to work or return to work in general practice in some capacity during your career break? Please feel free to add any further comments or ideas you would like to share*.

455 (38.2 %) participants to the main survey [2] provided free text comments; of these, 42 (9.2 %) made mention of appraisal and/or revalidation. These were managed using NVivo10. Two authors (RP and JD) separately coded the comments to explore emergent themes and together devised a coding framework to describe the thematic content using the method of constant comparison [17]. Verbatim quotes from the comments were selected to illustrate these themes.

## Results

Table 2 gives the characteristics of the 42 participants who mentioned appraisal and/or revalidation compared to the entire population who completed the main survey [2]. As shown, these respondents were older (76.2 % compared to 46.2 % aged 50 years and older), with more years’ experience in general practice (80.0 % compared to 48.0 % with more than 20 years’ experience), more often working as locums (17.1 % compared with 4.8 %) and more likely to express an intention to retire within the next 5 years (72.2 % compared to 41.9 %). They also rated rated revalidation as being ‘very important’ (63.3 %) to their intention to leave general practice within 5 years compared to those that had not commented on this topic (63.3 % compared to 14.9 %).

**Table 2:** Characteristics of the study sample compared to all those who participated in the main survey

	Study sample (*n* = 42) ^a^	Main survey (*n* = 1192) ^a^
Gender		
Male Female	25 (59.5 %) 17 (40.5 %)	622 (54.7 %) 515 (45.3 %)
Age (years)		
25–29 years 30–39 years 40–49 years 50–59 years 60–69 years 70 or more years	0 1 (2.4 %) 9 (21.4 %) 22 (52.4 %) 10 (23.8) 0	14 (1.2 %) 263 (23.0 %) 338 (29.6 %) 436 (38.2 %) 84 (7.4 %) 7 (0.6 %)
Main employment status		
GP contractor/principal Practice-employed salaried GP NHS trust-employed salaried GP Private sector-employed salaried GP Freelance GP (locum) Out-of-hours GP Academic GP	29 (70.7 %) 5 (12.2 %) 0 0 7 (17.1 %) 0 0	876 (74.9 %) 212 (18.1 %) 12 (1.0 %) 2 (0.2 %) 56 (4.8 %) 8 (0.7 %) 4 (0.3 %)
Length of time in general practice		
Less than 5 years 5–9 years 10–19 years 20–29 years 30 or more years	0 2 (5.0 %) 6 (15.0 %) 23 (57.5 %) 9 (22.5 %)	106 (9.0 %) 193 (16.3 %) 314 (26.6 %) 421 (35.6 %) 147 (12.4 %)
Intention to remain in practice general practice > 5 years		
Yes No	10 (27.8 %) 26 (72.2 %)	676 (58.1 %) 488 (41.9 %)

^a^ Percentages relate to the number who responded to each question

### Key themes

The key themes that emerged were the time-consuming and bureaucratic nature of the process; its relevance and validity to different work patterns (such as working as a locum); the emotional impact; the cumulative impact on retirement intentions; and the need for the process to be improved.

#### Bureaucratic and time-consuming

There was a widely expressed view that appraisal and revalidation was unnecessarily complex, time-consuming and a *“bureaucratic nightmare” (ID 50, age 50–59, male, principal)*. Some respondents supported the principle of appraisal and revalidation but described how the time taken to prepare for the process negatively impacts on their workload.

*“I must be one of the few GPs who support this [GP appraisal] in principle, I understand why it is necessary. But does the process have to be such an incredible meal! The time required to assemble the evidence is absurd and highly demotivating” (ID 153, age 50–59, female, principal).*

*“I have no problem with the principle of revalidation but find the whole process to be time consuming, gets in the way of my day to day GP work” (ID 253, age 50–59, male, principal)*

*“Appraisal good in theory and has benefits, but I reckon I’ve spent a week’s work on it this year” (ID 265, age 50–59, male, principal).*

Several comments linked the processes of revalidation to those of Care Quality Commission (CQC) and other inspections which were also seen as being overly complex. Together with other managerial and administrative activities faced by GPs, this was adding to the workload and work-related stress that many respondents described.

*“CQC is another Quango, which we end up paying for, as well as police checks, revalidation etc. etc. The list of bureaucratic nonsense is endless and demoralising” (ID 165, age 50–59, male, principal)*

*“At present patient based admin takes several hours a week, practice management takes several hours a week, yearly new contracts and enhanced services take several hours a week and keeping up to date and appraisal take several hours a week. This is impossible” (ID 106, age 50–59, female, principal)*

*“12–14 hour days is normal with e-mails and e-portfolio work for trainees and revalidation done in what’s left of the evenings and at weekends” (ID 243, age 50–59, female, principal)*

#### Relevance, validity and inequity

Many respondents felt that appraisal and revalidation lacked educational and developmental value, and hence served little purpose for the public or the profession. Collecting the required evidence was seen as having become a tick box exercise, and some respondents questioned the experience and skills of their appraiser to provide meaningful feedback.

*“The process was stressful and devoid of much real meaning. Tick box, limited scope for professionalism, over emphasis on legalism and managerialism….: this is a shame because they could be educational and an additional vehicle for professional development” (ID 306, age 70+, male, freelance GP).*

*“Complete waste of time. Everyone I speak to just does it to fulfil the legal requirements rather than for self-improvement which is what it should be for” (ID 385, age 50–59, male, practice-employed salaried)*

There was scepticism about the effectiveness of appraisal in improving standards of care.

*“A yearly appraisal is overkill in my opinion, expensive and unnecessary. I’m sure that Shipman would have passed with flying colours!” (ID 439, age 50–59, female, practice-employed salaried)*

*“Appraisal is a joke and insulting, I rarely have anyone appraise me who is as qualified to my level and I end up teaching them about something in medicine—it’s a farce and a waste of my time, if you pass easily why not have one every 5 years and concentrate on the ones in trouble” (ID 170, age 40–49, female, principal)*

Many comments reflected the way that appraisal, revalidation and other inspection, such as CQC, were experienced as irrelevant to general practice and demeaned their professionalism.

*“The degree of invasive scrutiny by the GMC, CQC, appraisals, revalidation is completely demeaning to a highly intelligent profession” (ID 385, age 50–59, male, practice-employed salaried)*

*“Am constantly having to prove that I am not a criminal or an incompetent to the CQC, GMC, NHS England—all treat us as the enemy, or as naughty children who cannot be trusted” (ID 72, age 50–59, male, principal)*

*“The CQC seems to me to be a costly and unwanted irrelevance which is growing out of control. Appraisal/validation could be made simpler. I have no evidence that either of the above has resulted in improvements in General Practice at all” (ID 256, age 60–69, male, principal)*

The perceived lack of flexibility in the requirements for appraisal and revalidation were seen as disadvantaging part-time, portfolio and locum GPs. This was felt to be limiting the extent to which GPs are being retained in the work force.

*“I know a large number of GPs who retired and did want to continue as part time but they found the appraisal process for part time or locums a heavy burden” (ID 25, age 60–69, male, locum)*

*“Inflexible working patterns by practices has meant that a mixed portfolio career incorporating Public Health with GP has not been attainable. Revalidation effectively excludes my achieving a mixed portfolio without a salaried post. Catch 22” (ID 4, age 60–69, male, locum)*

*“There are a lot of hurdles in revalidation which are more difficult for non-principals. Making it easier to do audits or get feedback might help” (ID 12, age 60–69, female, locum)*

#### Emotional consequence

The emotional impact of preparing for and undertaking appraisal and revalidation was a theme that was interpolated within many comments, as in the examples below.

*“Loathe most of the bureaucratic burden that has engulfed General Practice plus the thought of doing revalidation again is nauseating even though it wasn’t as bad as I feared” (ID 368, age 50–59, male, principal)*

*“…one of the most depressing parts of general practice is revalidation. It is so time-consuming and yet so unnecessary. It does not in any way improve my practice but it hugely negatively impacts on my time and my morale. I feel as though I’m being punished for one other doctor’s conduct in an inappropriate way” (ID 417, age 40–49, female, practice-employed salaried)*

*“The job is a relentless grind of patient demand, financial pressure, CQC inspections, and appraisals” (ID 171, age 50–59, male, principal)*

#### Cumulative impact

Reflecting the cumulative impact of the themes described above, several respondents described appraisal and revalidation as a final straw that was driving their decision to retire.

*“Appraisal nonsense final straw” (ID 80, age 50–59, male, principal)*

*“I am retiring in 4 months. Do not intend to revalidate which was the final straw after the messing up of the NHS superannuation scheme plus national pension changes” (ID 333, age 60–69, male, principal)*

*“APPRAISAL AT END OF CAREER IS A SURE FIRED WAY OF GPS THROWING THEIR TOWEL IN when they are still capable of helping in various clinical ways .........so I am getting out NOW and collecting my pension ASAP” (ID 210, age 50–59, male, principal)*

#### Improving the process

Several respondents highlighted the importance of changing appraisal and revalidation in order to make it less onerous, more flexible and more relevant to individual GPs’ pattern of work. In addition, it was felt that the processes of appraisal, revalidation, practice inspections by the CQC and the CCG could all be bundled into a single process which would reduce the cost and time involved.

*“Helping doctors do less paperwork for appraisal will really help them remain in UK and focus more on patient care and enjoy being a doctor” (ID 245, age 40–49, female, principal)*

*“Reduce scrutinisation of my work-contract monitoring/CQC/annual appraisal/revalidation” (ID 41, age 40–49, male, principal)*

*“Have only one evaluation of practice each year instead of separate inspections and data requirements by various agencies/bodies, such as Contract monitoring, CQC inspection, Practice visits by CCG, Health & Safety, Appraisal, Revalidation etc., etc.” (ID 98, age 60–69, male, principal)*

Specific elements of appraisal and revalidation, such as the expectation that an audit had been conducted, were seen as barriers to remaining in general practice. There was also a view that the extent of evidence needed to support appraisal and revalidation should be relevant to the stage of career that the doctor was at.

*“Flexible criteria for appraisals and revalidation to suit doctors with different levels of experience. One size fits all process should be stopped” (ID 59, age 60–69, male, principal)*

*“Revalidation yet again is too much at the end of one’s career. Regular appraisals, having done one revalidation slot, should suffice” (ID 382, age 50–59, female, practice-employed salaried)*

*“If I could be revalidated without having to undertake an audit, then I might consider staying on longer in a part time capacity” (ID 9, age 60–69, female, locum)*

## Discussion

Several themes emerged from the analysis of free-text comments about how appraisal and revalidation are influencing intentions to continue working as a GP: appraisal and revalidation as a bureaucratic, inflexible exercise that added to their already pressured workload; as an activity that has little educational value, relevance to professional development or quality of care; and as an issue that contributes to low morale, work-related stress and intentions to leave general practice. It was felt that appraisal failed to provide a formative context for personal development, and there was a lack of confidence that revalidation protected patients.

Several comments linked appraisal and revalidation together as part of an increasingly intrusive, overarching performance management process that included other processes, such as Care Quality Commission (CQC) inspections. These were described as being part of an overall de-professionalisation of general practice that is being driven by political goals and suspicions, as has been discussed elsewhere [18].

Although none of the comments mentioned appraisal as a process that supported remaining in the general practice workforce, this might be because of the overall emphasis of the main survey on reasons for leaving general practice or taking a career break.

Ways of improving appraisal and revalidation were suggested, in the main by simplifying requirements and increasing flexibility and developmental relevance to GPs’ varied working patterns and experience. This was seen as being especially important in order to prevent appraisal and revalidation acting as a barrier to continued working as a GP as in the later years of a career.

### Strengths and limitations

The themes identified should be interpreted in the context of the strengths and limitations of the main survey [2]. This had provided respondents with an opportunity to contribute free-text comments which enabled expression of views about how factors, such as appraisal and revalidation, contribute to the GP workforce crisis. However, there was uncertainty over the representativeness of respondents; responders may have been more dissatisfied or intent on leaving general practice than non-responders, although it is possible that those who felt the most over-worked or disillusioned may have been less motivated to respond and hence been under-represented. There was also evidence that they tended to be older, and were more often non-principals than GPs responding to the main survey.

There are inherent limitations in using free-text comments from questionnaire surveys [16]. Individuals vary in the extent to which they are inclined to contribute comments in questionnaires, reflecting issues such as lack of time and interest, and those who feel more critical or strongly about a topic are more likely to comment [16]. It is probable that further themes, including more positive ones, would have emerged had the questionnaire been specifically designed to seek views about appraisal and revalidation.

However, despite the findings being derived from data from only a small proportion of respondents, they should be interpreted in the context of the main survey which found that 28.6 % of respondents stating an intention to retire within the next 5 years rated their experience of revalidation as being important or very important to that decision. Hence, it seems likely that the views expressed here may reflect those of this group of GPs, and help to explain this finding.

### Implications

The themes reported here echo concerns voiced previously [19–22], and in the context of the workforce crisis facing general practice suggest a need for the processes involved in GP appraisal and revalidation to be reviewed. This appears to be particularly an issue for part-time GPs and those who are aged over 50 years and approaching the end of their career. The findings suggest that appraisal and revalidation is currently viewed by such individuals as inflexible, laborious and lacking relevance. There appears to be a need to tailor appraisal more closely to the stage of career that the GP has reached, as well as the varied activities undertaken by GPs with portfolio careers, locums, part time doctors and others who may find it harder to produce the currently required evidence. This might include greater flexibility in the frequency of appraisal and the requirements for different types of supporting evidence.

GPs are known to have more positive attitudes towards appraisal that has local ownership, is educationally oriented, and where the GP feels they have more control over the process [18]. Hence, appraisal could offer a mechanism to provide support and direction which might help to delay retirement [4]. However, this might require a substantial loosening of its link with performance management in order that GPs become more confident in appraisal as a formative process to support professional development.

Finally, there is a need for further research into GPs’ views as part of an overall evaluation of the costs and consequences of appraisal and revalidation. Such research should explore the aspects of appraisal/revalidation which are most problematic for GPs, investigate appraisers’ views and be aimed at identifying an approach that would be trusted and have greater effectiveness and applicability across the workforce. This might include reviewing evidence of the effectiveness of professional development, appraisal and revalidation systems in other health care systems to help identify an approach that is supportive to GPs while promoting the quality and safety of patient care.

## Conclusion

This study has identified ways in which the processes involved in appraisal and revalidation might be contributing to the workforce crisis that is facing general practice, but it is unclear how the views described here would be generalised to a more representative group of GPs. Rather than providing GPs with an opportunity for self-reflection that encourages non-judgemental recognition of strengths and professional development needs, its association with revalidation appears to be contributing to appraisal being viewed by at least some GPs with suspicion, scepticism and despair and is driving some GPs to seek early retirement.

Negativism about performance management is not limited to general practice. It is a global issue that is increasingly recognised in the business sector, with a trend among leading companies to move away from rigid review processes [23]. The reasons for this need to be explored, and the applicability to general practice considered. In conclusion, if GP appraisal and revalidation are to be retained, there appears to be an urgent need to review the current processes with the aim of establishing a valid, manageable, fair and trustworthy system that supports the development and retention of a high quality workforce within the NHS.

## Abbreviations

CCG, Clinical Commissioning Group; CQC, Care Quality Commission; GMC, General Medical Council; NHS, National Health Service; OOH, out of hours; RCGP, Royal College of General Practitioners
